# Physician shadowing by college students: what do patients think?

**DOI:** 10.1186/1756-0500-7-146

**Published:** 2014-03-14

**Authors:** Robert G Bing-You, Victoria M Hayes, Jennifer L Skolfield

**Affiliations:** 1 Maine Medical Center, 22 Bramhall St., Portland, ME 04102, USA

**Keywords:** Career choice, Shadowing, Premedical, Patient privacy, Confidentiality, Professional education

## Abstract

**Background:**

The objective of this study is to determine patients’ perceptions of physician shadowing by college students.

**Methods:**

Thirty-two patients who agreed to have a college student shadow their physician participated in semi-structured interviews during July and August 2013 at two outpatient family medicine centers. Qualitative techniques were utilized to analyze the transcripts of the patient interviews and identify common themes.

**Results:**

The majority of patients (78.1%) felt the college student had a neutral effect on their visit and denied having concerns about confidentiality (87.5%). No patient felt that having the college student present affected their ability to maintain a trusting relationship with their physician. Three themes emerged from the qualitative analysis: benefits to students, willing participation and sensitive issues. Most patients (78.5%) recognized that the student was in college or was a premedical student. The overwhelming majority of patients stated that they would have a college student shadow their physician again in the future.

**Conclusions:**

Despite concerns raised by other authors about the possible negative effects of physician shadowing by college students, this study shows that patients feel the impact to be primarily neutral and that there are many perceived benefits to both student and patient.

## Background

College students frequently shadow practicing physicians as a means to learn about careers in medicine. In this context, we use the term shadowing to reflect the activity of observing a physician doing patient care work and does not imply that the observer is conducting qualitative research. We often receive requests at our institution for such shadowing opportunities. Premedical students will list their shadowing activities on their applications to medical school as a way to show evidence of their interest and commitment to a medical career. Kitsis [1] recently raised several ethical concerns about this activity. Specifically, shadowing may breach physician’s fiduciary obligations to patients. Patient privacy and confidentiality may be violated. Patients may feel coerced by the attending physician to agree to have the student observe, or the reasons for shadowing may be misrepresented to patients.

What is the benefit of having college students shadow physicians? Larson et al. [2] described a summer program for high school students by the University of Oklahoma College of Medicine, which included shadowing experiences, and concluded the experience resulted in an increase likelihood of choosing a medical career. Hunter et al. [3] outlined a 5-day experience in the United Kingdom for 15–16 year old students and more than 70% of the students had a greater inclination to apply to medical school. Increasing the interest by college students in surgical careers through shadowing and other activities has been also reported [4,5].

Surprisingly, to our knowledge there have been no studies reporting patients’ perceptions of shadowing by college students. A recent review of the literature on physician shadowing did not report any such study [6]. Studies of patients’ perceptions of shadowing have focused on medical students [7-14]. In general, these studies have reported that patients are very positive about medical students shadowing, often for altruistic reasons, though patients can find the term medical student confusing [8], or feel aggrieved if they were not asked before participating [11].

In part because of the ethical concerns raised about this activity [1], and the lack of studies with premedical students, the objective of this study is to determine patients’ perceptions of college students shadowing physicians.

## Methods

### Design

This was a qualitative study using semi-structured interviews. The methodological orientation was a grounded theory approach.

### Setting and participants

The settings were the two Family Medicine Centers at Maine Medical Center. All of the attending physicians were asked if they would be willing to have a college student shadow them for one morning or afternoon. Eleven volunteered and only five were eventually needed. No specific parameters (e.g., the day of the week) were required of participation. A list of twelve college students came from either pre-medical advisors at local colleges or a group of college students who had recently expressed interest in an ongoing program called the Mini-Medical School, which is a week-long program sponsored by Maine Medical Center. All of the college students were asked if they would be interested in shadowing physicians and five agreed to participate. The college students completed confidentiality forms. No personal information of the physicians or the patients was collected and the Maine Medical Center Institutional Review Board exempted our project from review (IRB #4169X).

### Interview guide and procedures

The authors developed the interview guide based on the issues described in the literature about shadowing physicians (see the List of Semi-structured Interview Questions section below). On the day a college student was assigned to shadow a physician, the patients were informed by the medical assistant that a college student who was interested in medicine was present that day, shadowing their physician. They were also informed that another physician was conducting a study to explore patients' perceptions about college students shadowing their doctor. The patients were asked if they would be willing to have the college student present during their visit and also if they would be willing to answer some questions about the experience afterwards. They were all offered a written explanation of the study. No patients declined to have a college student shadow their Family Medicine physician. Four patients were not asked to participate due to urgent clinical matters (e.g., severe mental health issue) or inability to consent to participation (i.e., unaccompanied minor). While the college student observed, physicians conducted their usual clinical interaction with their patients (i.e., no changes were made during this activity because the student was present). If needed, patients were still physically examined in the typical and appropriate manner.

One of the authors, who is a physician, (VH) met with the patient immediately at the conclusion of their visit with the physician in the examination room. These semi-structured conversations lasted about ten minutes each. All of the interviews were audiotaped and transcribed. Thirty-two patients were interviewed between July and August 2013.

### Semi-structured interview questions

What was your experience of having a college student shadow your physician?

Did having the student affect the visit in a positive or negative way? Please describe how.

How do you think college students benefit by shadowing a physician?

Did you feel having the student present affected your ability to maintain a trusting relationship with your doctor?

What concerns, if any, did you have about confidentiality (e.g., not saying certain things to your doctor, or not asking certain questions, because the college student was present)?

How was it presented to you who this college student was and why they were there?

For what reasons did you agree to participate?

Would you do it again if the opportunity presented itself (i.e., have a college student shadow your physician for your visit)? Please explain your thoughts.

### Analysis

Qualitative techniques were used to analyze the transcripts [15]. Briefly, two of the authors (RB, VH) independently read all the transcripts, coding verbatim passages and establishing detailed codes. The commercial software program NVivo 10 was utilized for this coding process. These two authors subsequently reviewed their codes, and minimal differences were detected and resolved. Thirty-two codes were established. Both authors agreed that four of the interview questions had such minimal patient responses (i.e., essentially either yes or no responses only) that simple frequencies were calculated. The third author (JS) independently reviewed the transcripts and agreed with the results of the coding. All of the authors then jointly discussed and identified the themes that emerged by deciding which codes seemed to group naturally under a specific theme.

## Results

The majority of patients (n = 25, 78.1%) did not think the college student affected the visit in either a positive or negative way, whereas 21.9% thought the college student had a positive effect. No patient felt that having the college student present affected their ability to maintain a trusting relationship with their physician. The majority of patients (n = 28, 87.5%) had no concerns about confidentiality, whereas 12.5% had concerns (n = 3) or did not answer the question (n = 1). The three patients with concerns about confidentiality did not elaborate further.

Although all of the patients were informed that a college student would be shadowing their doctor today, only twenty-two of the twenty-eight patients (78.5%) who responded to the query whether they understood why the college student was there indicated they recognized the individual was a college student or some type of premedical student. A small number of patients (4/28, 14.3%) thought the individual shadowing was already in medical training (i.e., either medical student or intern). One patient thought the college student was a “student doctor” and only one patient indicated no remembrance of the background of the individual shadowing.

When the patients were asked if they would have a college student shadow their physician should the opportunity again present itself, the overwhelming majority (n = 31, 96.9%) stated they would (“*Yes. I think patients will benefit in the long run by allowing students to increase their knowledge this way*." "*Course*! *Yeah*, *sure. It was nice*.”). Only one patient answered “maybe” and no further explanation was given.

From the qualitative analysis of the interviews, three themes emerged: (1) benefits to students; (2) willing participation; and (3) sensitive issues.

“*I think they benefit greatly. They get to see real things going on and get themselves out of the classroom into the doctor*’*s office. Obviously*, *I*’*m a real person with real issues and she gets to see that. It all gets filed away collectively*, *I think*, *to bring back up some day when you need it. That*’*s all. It*’*s all experience*”.

“*One*, *she needs to learn. Two*, *I*’*m not against learning. And three*, *you know*, *I don*’*t think there are any secrets that I would have that she shouldn*’*t know about*”.

“…*people need to learn. And you need on*-*site training for certain professions. I think they absolutely should be able to come in and observe and learn*”.

“*I think it definitely benefits them because they can see things hands*-*on how they*’*re happening instead of reading about it or hearing about it*”.

“*I think there*’*s nothing like hands*-*on learning to me*, *but that*’*s the way I learned*, *so*…*I think it can only benefit them because textbooks are never going to put you in a situation like real life*”.

1. *Benefits to students*. Many patients thought that the college students benefited from having the opportunity to observe and learn. They noted the “hands-on” experience outside of a classroom, even though just observing, was valuable.

Several patients thought that shadowing helped the college students decide about going into a career in medicine (“*If he wants to think about possibly going into medicine*, *it helps him see more what the daily routine is like. It can help him decide*.”) and that the patients themselves were helping the profession recruit future physicians (“*Doctors are here to do whatever doctors are here to do and whatever I can do to help that process is good. We probably need more doctors down the road. So*, *hey*, *good for a young person looking to get into medicine*.” “*I have no problems helping the medical community continue on to get new volunteers or new recruits. I work in a business that is always looking to recruit as well*.”).

Even though these were only college students, patients felt that this experience would help the college students be a better physician someday.

“*I think it*’*s important*! *It was going to help her become a better doctor*”.

“*I think I*’*m hopefully helping to make a better doctor down the road*”.

“*It doesn*’*t bother me because my mother is a retired R.N. so how else are people going to learn*? *They*’*re the next generation of people who could be taking care of me. I think it*’*s part of training*”.

“*For the benefit of the student*, *I was willing*”.

“*If having her here for my visit helps her and her future*, *then good*”.

2. *Willing participation*. Many patients very willingly participated in having a college student observe from an altruistic perspective.

Patients who had previous interactions with learners, though not necessarily students who were shadowing, expressed this as reason to participate (“*When I saw Dr*. ___ *in November in her other office*, *I had a student evaluate me initially and so I*’*m perfectly comfortable with it. I also had a student at my physical therapy session recently too*.” “*It*’*s like if you go to a hospital*, *especially in Boston where I*’*m from originally*, *at teaching hospitals you*’*ll have a group of half a dozen. A doctor with numerous interns or residents who accompany them around and it*’*s fine*.”).

Those patients who have been in a learning role themselves indicated this was a reason they allowed the college students to shadow (“*It*’*s always nice to see a student. I appreciate them. I was a student not too long ago*, *so*…*why not*.” “*I*’*m used to students working in the hospital and I was a student myself once upon a time and I was always appreciative of all the experiences I could get and the people that allowed me to be a part*.”).

“*If I had to have a GYN exam*, *I would have a woman doctor. Yeah*, *and then she could have someone come in unless it was a guy. That would bother me*”.

“*Geez*, *I hate to even bring this up*, *but with my being a man and the student being a female*, *this stuff*, *normal physical stuff doesn*’*t bother me at all*, *but once again*, *taking my socks off*, *if I had to show my foot to her*, *that might have been a little intimidating*”.

3. *Sensitive issues*. Although the vast majority of patients’ comments were positive about having college students shadow during the visit with their physician, a few patients raised a theme about potential sensitive issues. One aspect was being uncomfortable depending on the gender of the college student.

Other patients noted sensitivity around privacy matters (“*Well*, *I wouldn*'*t want her to look when I was being checked by the doctor in that personal place*, *you know*." "*I wouldn*'*t want to talk about my sex life in front of her*, *but other than that*." "*If I had a kind of touchy subject*, *I would say I prefer a one*-*on*-*one with my doctor*.").

## Discussion

We have described in this qualitative study the perceptions of patients who had a college student shadow during a visit with their physician. We found that the majority of these patients felt the college student had a neutral effect on the visit, and had no concerns about confidentiality. These perceptions run counter to the concerns raised by previous authors [1,16]. No patient in this study felt that having the college student present affected their ability to maintain a trusting relationship with their physician and therefore the patient-doctor relationship was preserved and not strained [16].

These patients perceived several benefits for the shadowing students. They recognized the value in getting beyond the textbooks and classrooms and learning from actual experiences. These patients also supported the notion that shadowing can help promote an interest in a medical career [2,3], and relished having a role in recruiting future physicians. Even though these patients recognized that these were college students, many hoped the shadowing experience would help them be a better doctor someday should they enter the medical field. Patients have a strong belief in the importance of teaching [14].

The patients in this study were very willing participants and no patient declined when given the option. This reflects a strong altruistic inclination by these patients. Although patients have no obligation to participate in medical student teaching [16] or college student shadowing, they may be unselfish in wishing to help the college student even though they are seeing their physician for their medical needs. In a study with second-year medical students, Stacy et al. [13] described potential patient benefits in addition to feelings of altruism and satisfaction from helping (e.g., gains from talking about their problems, learning more about their condition because of the learning encounter). Of note is that willingness to participate was also enhanced by prior learner interactions and if the patients had been in similar learning roles themselves.

Even though the vast majority of these patients were very willing participants and perceived many benefits to the students, we thought it was important to report themes, if identified, related to potential sensitive issues. Others have also highlighted female patients being uncomfortable with male learners [7]. Patients may have individualized private matters which they would not want to share in front of a college student (e.g., sensitive parts of the history such as sexual concerns, or intimate parts of the physical exam). Future studies could delve further into the patient’s experience when very sensitive issues are involved.

Kitsis [1] raised the concern about misrepresentation about shadowing and Chipp et al. [8] described patients being confused about student terms or titles. The majority of the patients in this study recognized the individual shadowing was a college student. This obviously could reflect our preparation of the patient when asking for permission to participate. Even so, a few patients were confused and thought the student was already in medical training. We support the recommendations by Howe et al. [17] to empower patients with adequate information about the encounter and by Kitsis et al. [6] to develop a handbook on physician shadowing, which could include a code of conduct, patient consent form, and privacy/confidentiality agreements.

The results of our qualitative analysis have certain limitations. Only Family Medicine physicians were shadowed, and it is possible patients seeing other specialties would have a different perspective. Our findings are from one institution, which has a long academic tradition in the community (i.e., having medical students and residents present). As the interviewer was a physician, it is possible patients may have given different responses to a non-physician due to a perceived power differential. Patients visiting physicians in private practice may feel less inclined to have college students shadow. Outpatient versus inpatient settings, or settings with no expected long-term relationships with physicians (e.g., Emergency Room), may have different findings. Future research could focus on the relationship of prior shadowing experiences with any impact on medical school success or outcomes.

## Conclusions

We conclude that despite concerns raised by other authors about the possible negative effects of physician shadowing by college students, this study shows that these patients feel the impact to be neutral or positive and that there are many perceived benefits to both student and patient. Patients’ sense of altruism was not offended [16] and the overwhelming majority would have a college student shadow again when the opportunity arose.

## Competing interests

The authors declare that they have no competing interests.

## Authors’ contributions

All of the authors participated in the design of the study. VH interviewed the patients. VH and RBY coded the transcripts. All of the authors developed the major themes from the qualitative analysis, and read and approved the final manuscript.
